# Conditional survival analysis and dynamic prediction of long-term survival in Merkel cell carcinoma patients

**DOI:** 10.3389/fmed.2024.1354439

**Published:** 2024-02-08

**Authors:** Jin Zhang, Yang Xiang, Jiqiu Chen, Lei Liu, Jian Jin, Shihui Zhu

**Affiliations:** ^1^The First Affiliated Hospital of the Naval Medical University, Shanghai, China; ^2^Shanghai Children’s Medical Center, School of Medicine, Shanghai Jiaotong University, Shanghai, China

**Keywords:** Merkel cell carcinoma, MCC, conditional survival, overall survival, SEER, nomogram

## Abstract

**Background:**

Merkel cell carcinoma (MCC) is a rare type of invasive neuroendocrine skin malignancy with high mortality. However, with years of follow-up, what is the actual survival rate and how can we continually assess an individual’s prognosis? The purpose of this study was to estimate conditional survival (CS) for MCC patients and establish a novel CS-based nomogram model.

**Methods:**

This study collected MCC patients from the Surveillance, Epidemiology, and End Results (SEER) database and divided these patients into training and validation groups at the ratio of 7:3. CS refers to the probability of survival for a specific timeframe (y years), based on the patient’s survival after the initial diagnosis (x years). Then, we attempted to describe the CS pattern of MCCs. The Least absolute shrinkage and selection operator (LASSO) regression was employed to screen predictive factors. The Multivariate Cox regression analysis was applied to demonstrate these predictors’ effect on overall survival and establish a novel CS-based nomogram.

**Results:**

A total of 3,843 MCC patients were extracted from the SEER database. Analysis of the CS revealed that the 7-year survival rate of MCC patients progressively increased with each subsequent year of survival. The rates progressed from an initial 41–50%, 61, 70, 78, 85%, and finally to 93%. And the improvement of survival rate was nonlinear. The LASSO regression identified five predictors including patient age, sex, AJCC stage, surgery and radiotherapy as predictors for CS-nomogram development. And this novel survival prediction model was successfully validated with good predictive performance.

**Conclusion:**

CS of MCC patients was dynamic and increased with time since the initial diagnosis. Our newly established CS-based nomogram can provide a dynamic estimate of survival, which has implications for follow-up guidelines and survivorship planning, enabling clinicians to guide treatment for these patients better.

## Introduction

Merkel cell carcinoma (MCC) is a rare invasive neuroendocrine skin malignancy with a tendency to lymphatic spread (1–4). In the United States, the incidence of MCC has steadily increased from 0.6 per 100,000 person-years in 2013 to 0.7 in 2019 (1). The rising trend of MCC incidence is worrisome due to its high mortality rate compared to other skin malignancies which partially due to its advanced stage diagnoses (5–7). The clinical presentation of this disease lacks specificity and may result in unfavorable prognostic outcomes (8). Approximately 30% of diagnosed patients present with local or lymph node metastasis at the time of initial diagnosis (3, 9).

Current treatment guidelines typically use TNM classification for initial staging (9). Generally, surgical removal of the lesion is the preferred treatment option (8, 9). In case of the advanced disease, radiotherapy, chemotherapy, and immunotherapy may be utilized (3, 8, 9). Because of delayed diagnosis, a high incidence of metastasis, and a low survival rate of this disease, despite the development of standardized treatment protocols due to increased incidence and knowledge, research on the long-term outcomes of MCC patients remains insufficient (7, 9). Furthermore, to enhance prognostic accuracy and improve treatment efficacy, it is crucial to have a comprehensive understanding of therapeutic strategies. As a result, we must dynamically evaluate the prognosis of patients to guide our treatment plan and follow-up strategy, thereby optimizing the clinical management of this particular tumor.

Recently, conditional survival (CS) assessment has become an additional means of dynamically determining the prognosis of cancer survivors (10, 11). CS estimation can offer more precise prognostic data to clinicians and patients, but it fails to take into account the clinicopathological characteristics of patients (12). Although many assessment tools have been developed for prognosis prediction in MCC patients, none of them have focused on changes in prognosis over time. Moreover, the traditional nomograms do not provide dynamic prognostic information over the course of survival time, despite allowing for personalized predictions based on the clinicopathological features of patients (13). Therefore, in this study, we combined the CS analysis and nomogram model to develop a novel CS-based nomogram for dynamic estimation of MCC’s outcomes.

The purpose of this study is to examine the CS pattern in MCC patients using the SEER database from the United States and create a CS-nomogram model that can offer clinicians and patients with personalized and up-to-date prognostic data.

## Methods

### Patients and variables selection

This study collected 3,843 patients in the SEER database diagnosed with MCC between 2000 and 2019 (14). The International Classification of Diseases for Oncology (ICD O-3) site recode was MCC with histology codes 8247/3. We have registered a SEER account and obtained approval of using data for research purposes, which has been ethically approved. The demographics and clinical characteristics including age at diagnosis, sex, race, marital status, household income, rural/urban residence, tumor site, tumor size, AJCC stage, multiple primary tumors, lymph nodes involved, surgery, radiotherapy, and chemotherapy were obtained for MCC patients. We excluded the patients with the following criteria: (1) AJCC stage unknown; (2) Not the first primary malignancy tumor; (3) Lack of essential variables; (4) Survival month unknown or < l month. The clinical endpoint of this study is patient overall survival (OS), which defined as the time interval between the diagnosis to death or final follow-up regardless of cause. OS analysis was estimated through the Kaplan–Meier method.

### Statistical analysis

This study partitioned the chosen MCC patients into a training set and a validation set at a ratio of 7:3. Subsequently, we computed the total count and percentage of categorical variables in the entire cohort, training cohort, and validation cohort.

CS(y|x) is the probability of additional y years of survival given that the patient has not died of MCC by a specific period of time (x years) after initial diagnosis (15). Standard definition of conditional probability was used to calculate CS: CS(y|x) = OS(y + x)/OS(x). The estimation of survival probability for x- and (x + y)-years was conducted via the Kaplan–Meier methods by using OS(x) and OS(y + x), respectively. For instance, if we aim to estimate the conditional survival (CS) rate of patients who have survived for 2 years after the initial diagnosis, for three more years, we can calculate CS(3|2) as CS(3|2) = OS(3 + 2)/OS(2) = the 5-year OS rate divided by the 2-year OS rate. Additionally, we conducted Kernel-smoothing hazard function analysis to examine the mortality of MCC patients within 1 year of follow-up (16).

In this study, we utilized the least absolute shrinkage and selection operator (LASSO) regression technique with 10-fold cross-validation to identify independently prognostic factors in the training cohort (17). Subsequently, multivariate Cox regression was conducted to confirm the prognostic value of the selected variables and integrate them into a new nomogram model (18). The CS concept was finally utilized in the development of a CS-nomogram, which is capable of providing personalized, dynamic prognostic information that is continually updated. It incorporates patient risk assessment to determine individual survival and CS rates. This approach offers a more objective and accurate method for predicting patient outcome.

The probability of 3-, 5-year, 7-year OS and 7-year CS could be estimated with our nomogram model. The accuracy of the CS-nomogram was evaluated through calibration curves, where a curve closer to the 45° line indicated higher accuracy. We also used the concordance index (C-index) and the receiver operating characteristic (ROC) curves with area under the curve (AUC) to assess the model’s discrimination and stability. Furthermore, the clinical applicability of the CS-nomogram was validated through decision curve analysis (DCA), which measured the net benefit of medical intervention. These analytical methods provide precise and objective measures to evaluate the performance and utility of the CS-nomogram.

Statistical tests were conducted with a two-sided approach and a statistical significance threshold of *p* < 0.05. All statistical analyses were performed using R (version 4.1.0).

## Results

### Demographic and clinicopathological characteristics

A total of 3,843 patients in the SEER database were diagnosed with MCC between the years 2000 and 2019 (Table 1). The patients were divided into a training group (*n* = 2,690) and a validation group (*n* = 1,153). The majority of individuals in the whole cohort were aged over 60 years (87.5%), identified as white race (95.9%), and resided in a metropolitan county (87.9%) at the time of diagnosis. In terms of tumor characteristics, a majority of primary sites were located in the head, neck, and face (36.4%) or in extremities (47.6%). Moreover, 67.0% of patients exhibited negative lymph nodes. And 44.7% of all patients were classified as being in AJCC stage I, 18.8% in stage II, 30.7% in stage III, and 5.8% in stage IV. In terms of treatment, 3,472 patients (90.3%) underwent surgical intervention, 2,120 patients (55.2%) received radiotherapy, and chemotherapy was not administered to 3,385 patients (88.1%).

**Table 1:** Clinicopathologic characteristics of MCC.

Parameters	Total cohort (*N* = 3,843)	Training cohort (*N* = 2,690)	Testing group (*N* = 1,153)
Age at diagnosis			
≤60	479 (12.5%)	333 (12.4%)	146 (12.7%)
61–70	831 (21.6%)	584 (21.7%)	247 (21.4%)
71–80	1,235 (32.1%)	869 (32.3%)	366 (31.7%)
>80	1,298 (33.8%)	904 (33.6%)	394 (34.2%)
Sex			
Male	2,401 (62.5%)	1,688 (62.8%)	713 (61.8%)
Female	1,442 (37.5%)	1,002 (37.2%)	440 (38.2%)
Marital status			
Single	1,348 (35.1%)	936 (34.8%)	412 (35.7%)
Married	2,287 (59.5%)	1,607 (59.7%)	680 (59.0%)
Unknown	208 (5.4%)	147 (5.5%)	61 (5.3%)
Race			
White	3,684 (95.9%)	2,576 (95.8%)	1,108 (96.1%)
Nonwhite	159 (4.1%)	114 (4.2%)	45 (3.9%)
Tumor site			
Head, neck, and face	1,398 (36.4%)	985 (36.6%)	413 (35.8%)
Trunk	431 (11.2%)	314 (11.7%)	117 (10.1%)
Extremity	1831 (47.6%)	1,270 (47.2%)	561 (48.7%)
Skin NOS	183 (4.8%)	121 (4.5%)	62 (5.4%)
Multiple primary tumors			
No	2,183 (56.8%)	1,523 (56.6%)	660 (57.2%)
Yes	1,660 (43.2%)	1,167 (43.4%)	493 (42.8%)
Tumor size			
<40 mm	1841 (47.9%)	1,289 (47.9%)	552 (47.9%)
≥40 mm	2002 (52.1%)	1,401 (52.1%)	601 (52.1%)
AJCC stage			
I	1719 (44.7%)	1,186 (44.1%)	533 (46.2%)
II	722 (18.8%)	512 (19.0%)	210 (18.2%)
III	1,178 (30.7%)	834 (31.0%)	344 (29.8%)
IV	224 (5.8%)	158 (5.9%)	66 (5.7%)
Lymph nodes involved			
No	2,573 (67.0%)	1794 (66.7%)	779 (67.6%)
Yes	1,270 (33.0%)	896 (33.3%)	374 (32.4%)
Surgery			
No	371 (9.7%)	255 (9.5%)	116 (10.1%)
Yes	3,472 (90.3%)	2,435 (90.5%)	1,037 (89.9%)
Radiotherapy			
No	1723 (44.8%)	1,187 (44.1%)	536 (46.5%)
Yes	2,120 (55.2%)	1,503 (55.9%)	617 (53.5%)
Chemotherapy			
No	3,385 (88.1%)	2,363 (87.8%)	1,022 (88.6%)
Yes	458 (11.9%)	327 (12.2%)	131 (11.4%)
Household income			
<65,000$	1,609 (41.9%)	1,137 (42.3%)	472 (40.9%)
≥65,000$	2,234 (58.1%)	1,553 (57.7%)	681 (59.1%)
Rural–urban			
Non-metropolitan	465 (12.1%)	333 (12.4%)	132 (11.4%)
Metropolitan	3,378 (87.9%)	2,357 (87.6%)	1,021 (88.6%)

NOS, not other specific.

### Conditional survival analysis of MCC

**Figure 1:**
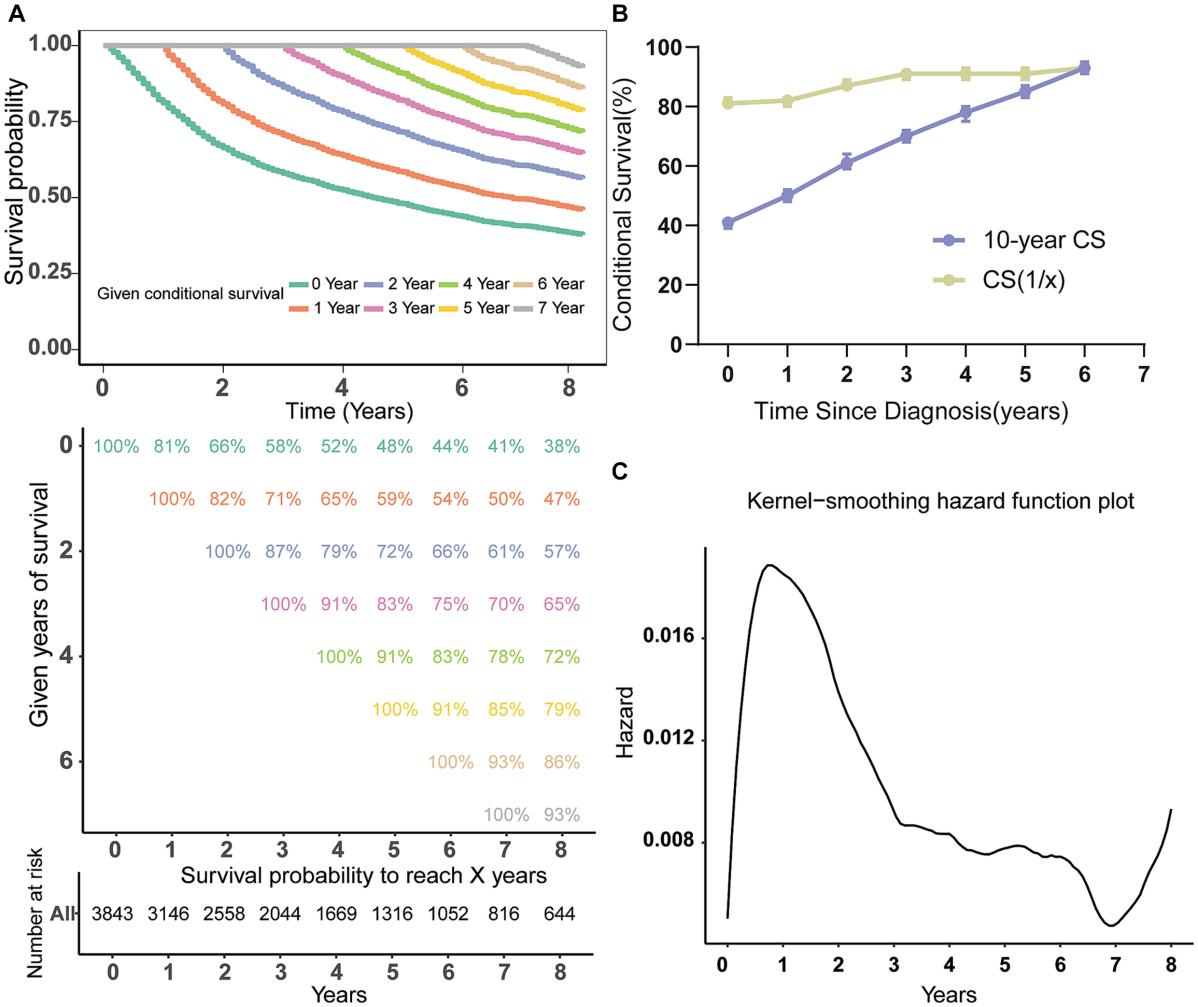
Conditional survival analysis of patients with MCC. **(A)** Kaplan–Meier estimates of survival at diagnosis (0 years) and conditional survival based on years already survived after diagnosis (1–7 years). **(B)** CS(1|x) curve demonstrated the probability of survival additional 1 year after surviving for x years since diagnosis and 7-year CS curve showing the 7th year of survival after surviving for x years since diagnosis; **(C)** the Kernel-smoothing hazard function curve. CS, conditional survival.

The study utilized the Kaplan–Meier technique to examine the OS likelihood of individuals with MCC from the SEER database. The findings indicated that MCC patients had a 58, 48, and 41% chance of survival at 3, 5, and 7 years of follow-up, correspondingly (Figure 1A). Through the use of CS analysis, we have identified a noteworthy increase in the 7-year survival rates among patients who have been survived for additional years. The initial rate began at 41%, and then increased to 50, 61, 70, 78, and 85% before culminating in an impressive 93%. It is important to note that the improvement in survival rates over time was not a linear progression. The (1 | x) curve displays the survival rate of patients in the second year following x years since diagnosis, revealing a lower second-year survival rate for patients who have survived 1 or 2 years after diagnosis (Figure 1B). Furthermore, the annual hazard curve presented the greatest probability of death among MCC patients during the first year following diagnosis (Figure 1C).

### The CS-nomogram development and validation

**Figure 2:**
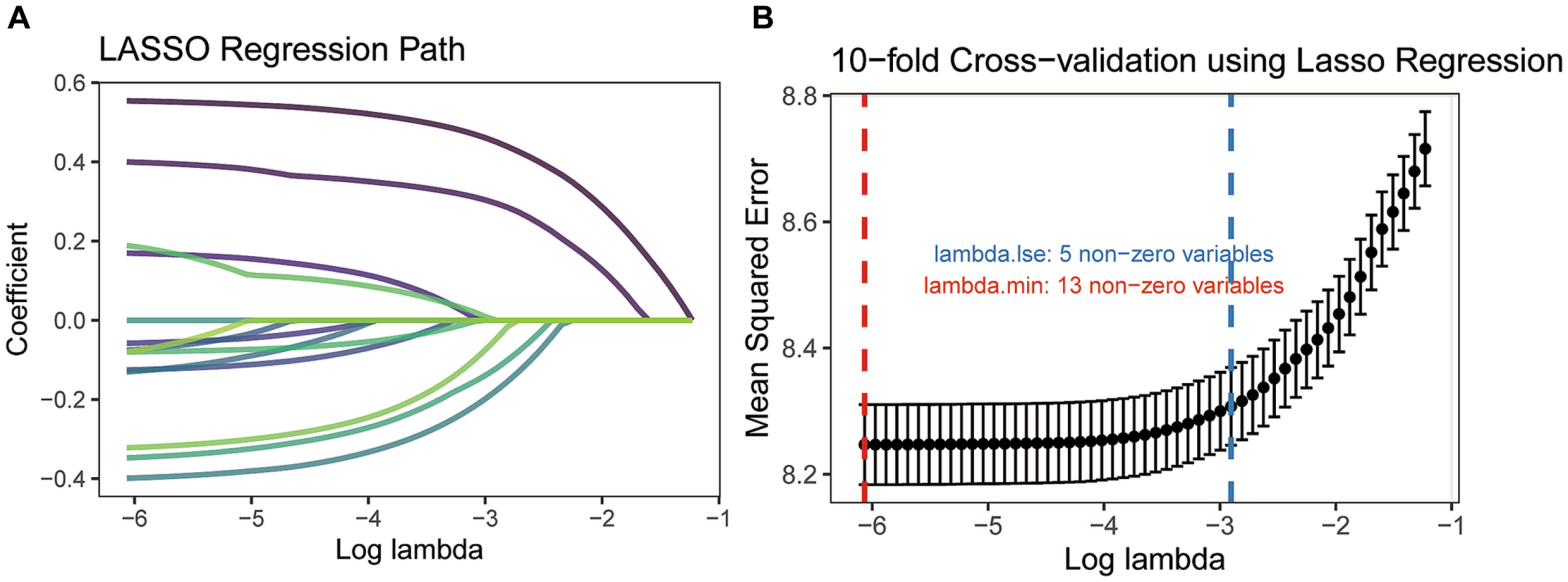
The least absolute shrinkage and selection operator (LASSO) regression analysis **(A)** and 10-fold cross-validation **(B)**.

**Figure 3:**
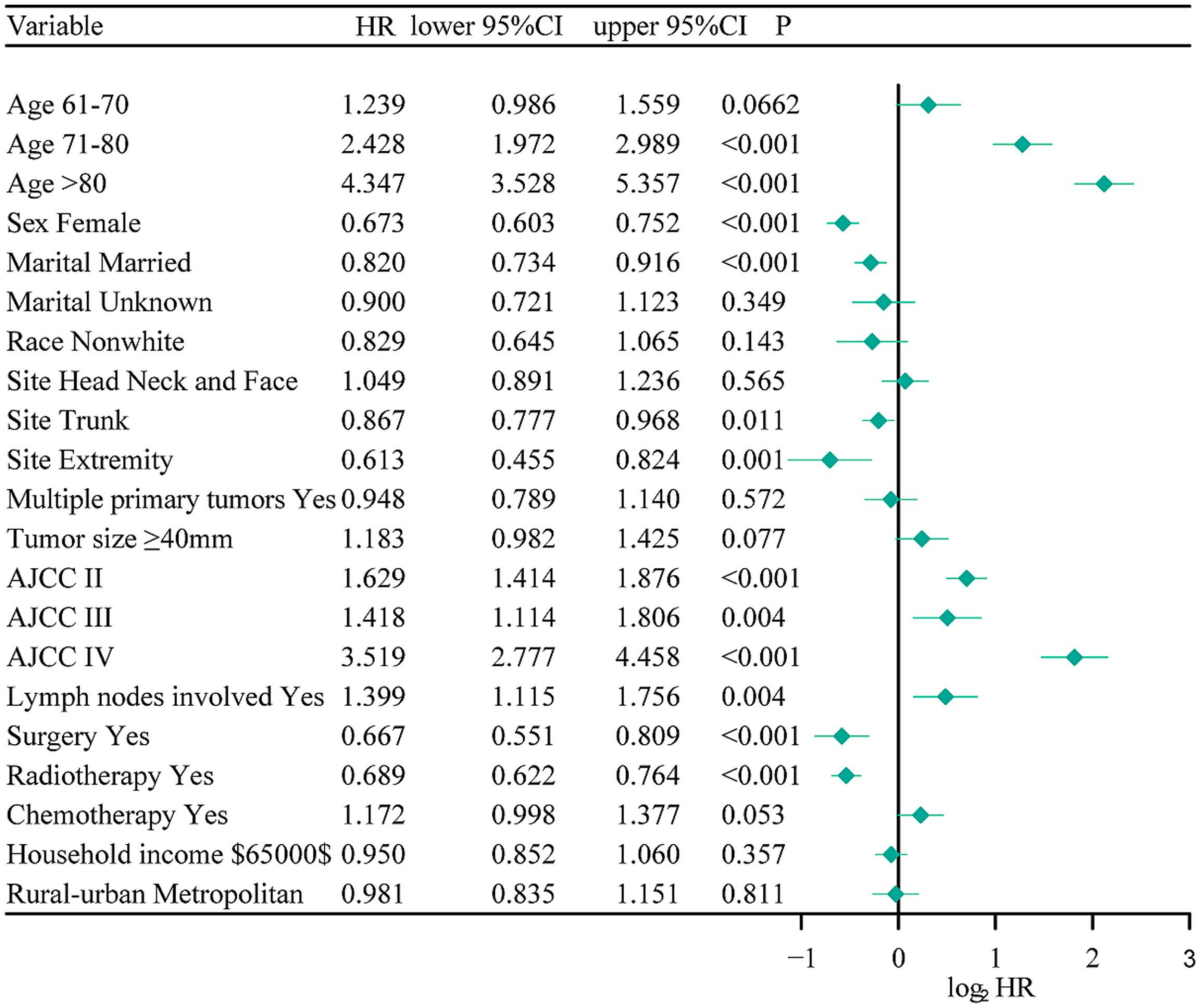
The multivariate Cox regression forest plot. NOS, not other specific; RT, radiotherapy; CT, chemotherapy.

**Figure 4:**
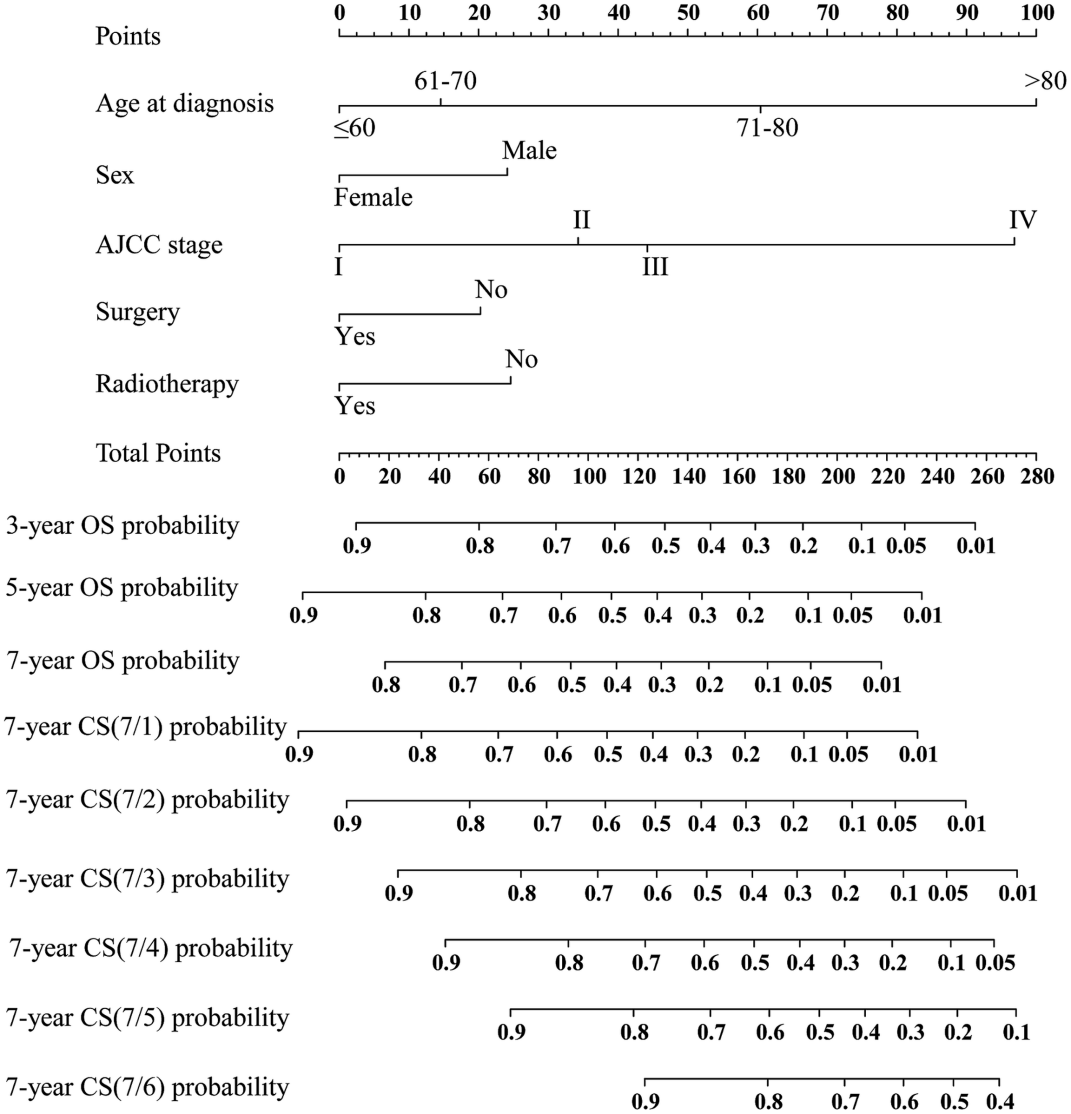
Conditional survival-based nomogram predicting 3-, 5-, and 7-year overall survival (OS) and 7-year conditional survival (CS) for MCC patients. NOS, not other specific.

Based on the training cohort, the LASSO regression model, with 10-fold cross-validation, ultimately selected 5 significant predictors for developing the prognosis prediction model in MCC patients (Figures 2A,B). These predictors consisted of age at diagnosis, sex, AJCC stage, surgery, and radiotherapy. Additionally, the prognostic indicating value of these factors in MCC patients was further confirmed through the use of a multivariate Cox regression forest plot (Figure 3) with a highly significant probability *p* < 0.05. Subsequently, we have successfully created a dynamic CS-nomogram that could continuously update the 3, 5, and 7-year OS rates, as well as the 7-year CS survival rates, for patients with MCC (Figure 4).

**Figure 5:**
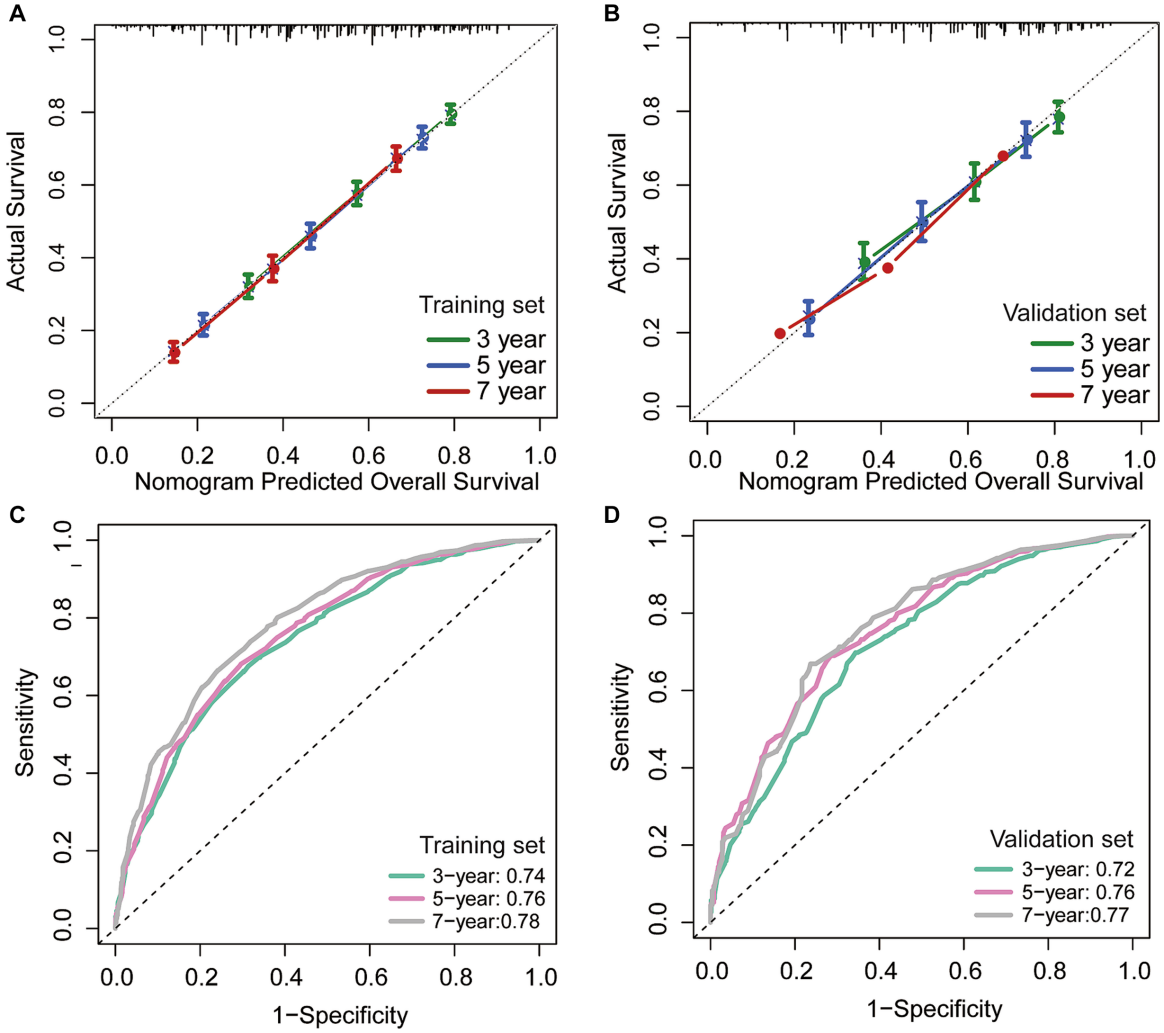
The CS-nomogram validation. Calibration plots of the nomogram for training and **(A)** validation group **(B)**; time-dependent area under curve (AUC) curves for training **(C)** and validation group **(D)**. AUC, area under the curve.

**Figure 6:**
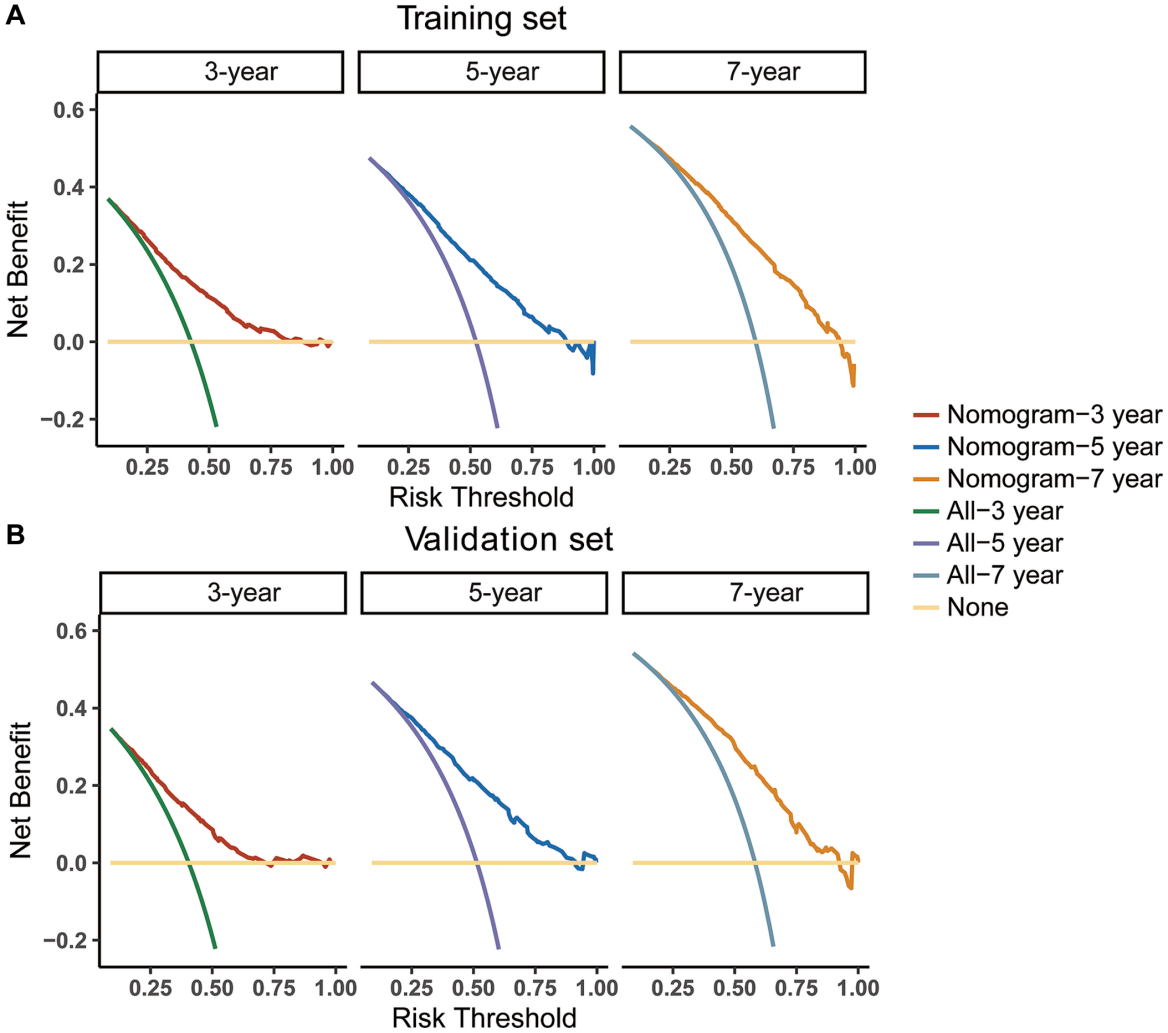
Decision curve analysis (DCA) curves for evaluating clinical usefulness of the nomogram in training **(A)** and validation group **(B)**.

In addition, we utilized an internal validation group of 1,153 patients from the SEER database to confirm the accuracy of our nomogram. Our innovative model exhibited favorable predictive value with a C-index of 0.692. We assessed the accuracy of the CS-nomogram in both the training and validation cohorts by using calibration plots, which demonstrated excellent consistency between the CS-nomogram’s predictions and actual 3, 5, and 7-year OS outcomes, with curves closely resembling the 45-degree line (Figures 5A,B). The ROC analysis at 3, 5, and 7-year OS outcomes revealed that this model has strong discrimination. The AUC values for 3, 5, and 7-year OS outcomes were 0.74, 0.76, and 0.78 in the training set, and 0.72, 0.76, and 0.77 in the validation set (Figures 5C,D). Additionally, the DCA curves indicated that the CS-nomogram has potential as a useful tool for guiding medical intervention (Figure 6).

## Discussion

MCC is a rare malignant neuroendocrine tumor of the skin (1, 2). The incidence of MCC tends to increase with advancing age and the number of diagnosed cases has also risen in recent years (19). Despite ongoing research, the etiology of MCC remains unclear, posing significant challenges for patients, physicians, and researchers in the diagnosis of this malignancy (2, 8, 20). Additionally, the overall 5-year relative survival rate of MCC patients is relatively low, highlighting the need for accurate prognostic factors and survival estimation for effective management and monitoring (3, 21, 22). In this regard, our research seeks to establish a dynamic nomogram model based on CS for personalized and dynamic survival prediction. Through a nationwide study utilizing the SEER database, we have developed a survival prediction model specifically focused on the CS of patients with MCC, aimed at enhancing our comprehension of the tumor’s prognosis.

In recent times, CS analysis has emerged as a novel technique for evaluating cancer survival, with distinct advantages in anticipating poor prognosis cancer and determining changes in survival rates (23, 24). Presently, numerous studies are investigating the practical application of this method in the clinical setting (25, 26). This study initially examined the CS pattern of MCC patients and found that their survival improved dynamically with each passing year survived. Meanwhile, this improvement was non-linear and slowest in the 1st year after diagnosis. The annual hazard curve additionally indicated that the mortality rate is highest within the initial year post-diagnosis, emphasizing the critical significance of closely monitoring patients during the initial year of diagnosis. In light of this crucial time period that we discovered, this article emphasized the necessity of implementing proactive treatment approaches and devising a comprehensive follow-up program at the initial stages of treatment. By strengthening treatment strategies and optimizing treatment outcomes, the survival prospects of MCC patients can be bolstered. Therefore, the precise and timely forecasting of survival is paramount. CS analysis can aid physicians and patients in gaining a better understanding of the patient’s prognosis and treatment process, while also providing a foundation for designing treatment plans and follow-up procedures (27).

Traditional methods for survival analysis have limitations when applied to real-time data (10, 20, 28). To address this issue, this study utilized CS analysis to improve the dynamic survival estimation capability of the nomogram model. As a result, a new and updated CS-nomogram was developed (10), which provides dynamic survival information for MCC survivors. Our model enables us to identify high-risk patients and enhance the treatment process by providing dynamic prognosis evaluation. Furthermore, through the use of techniques like the C-index, calibration plot, ROC analysis, and DCA analysis (10, 29, 30), we have verified the outstanding predictive capabilities and practical value of our newly developed CS-nomogram model. As a result, the CS-based nomogram offers numerous advantages over conventional methods, particularly in terms of its ability to provide dynamic responses in survival analysis. The model’s versatility gives it significant potential for widespread application, and it is poised to become a vital tool for predicting outcomes and evaluating treatment effectiveness in MCC cases. Ultimately, this will help improve the accuracy and reliability of predictions, promoting better patient outcomes and a higher quality of life.

In spite of medical advances, patients with MCC still struggle to find better survival rates (31, 32). To address this, it is crucial to conduct clinical trials. Our research found that MCC patients have a high risk of mortality within a specific time frame after diagnosis, which highlights the need for intensified supportive care during this period. Additionally, our CS-based nomogram model can provide risk classification for patients, aiding in better clinical trial enrollment.

There are still limitations to this study. Firstly, due to its retrospective nature, selection bias is inevitable. Secondly, our prognostic risk factors are insufficient. If pathology-related features, detailed treatment information, patient comorbidities and other important information were included in the model for analysis, the prediction of the nomogram would be more accurate and tailored. Thirdly, our nomogram model requires further external validation. Fourthly, due to the inherent limitation of the SEER database, detailed information on treatments such as radiotherapy type, radiation dose, together with the choice of chemotherapeutic agents were unavailable. Moreover, Avelumab therapy has dramatically improved the prognosis of metastatic MCC in recent years, due to the lack of relevant information on Avelumab application, we are unable to rectify this, potentially leading to a bias. Future research would necessitate the utilization of higher quality multi-centric data that could help identify potentially prognostic factors, thereby refining our model and its predictive capabilities. Finally, as treatment strategies improve, this CS nomogram is expected to be regularly updated.

## Conclusion

The CS analysis conducted in this study highlighted the dynamic and fluctuating nature of survival rates among patients with MCC. After applying LASSO regression and multivariable Cox regression, five predictive factors were selected, and a novel CS-nomogram was constructed with excellent performance. This model provides reliable prognostic information to help optimize clinical decisions by providing dynamic updates on prognosis. However, future studies will require prospective data and additional predictors to validate and update the model, making it more applicable in more generalizable settings.

## Data availability statement

The datasets presented in this study can be found in online repositories. The names of the repository/repositories and accession number(s) can be found in the article/supplementary material.

## Author contributions

JZ: Methodology, Software, Supervision, Validation, Visualization, Writing – original draft, Writing – review & editing. YX: Methodology, Software, Supervision, Validation, Visualization, Writing – original draft, Writing – review & editing. JC: Formal analysis, Writing – review & editing. LL: Formal analysis, Writing – review & editing. JJ: Data curation, Validation, Writing – review & editing. SZ: Funding acquisition, Supervision, Writing – review & editing.
